# Effect of Milling Time and Reinforcement Volume Fraction on Microstructure and Mechanical Properties of SiC_p_-Reinforced AA2017 Composite Powder Produced by High-Energy Ball Milling

**DOI:** 10.3390/ma17020435

**Published:** 2024-01-16

**Authors:** Shimelis Bihon Gasha, Maik Trautmann, Guntram Wagner

**Affiliations:** Professorship of Composites and Material Compounds, Institute of Materials Science and Engineering (IWW), Chemnitz University of Technology, 09125 Chemnitz, Germany; maik.trautmann@mb.tu-chemnitz.de (M.T.); guntram.wagner@mb.tu-chemnitz.de (G.W.)

**Keywords:** composite powder, high-energy ball milling, crystallite size, lattice strain, microhardness

## Abstract

The influence of milling time and volume fraction of reinforcement on the morphology, microstructure, and mechanical behaviors of SiC_p_-reinforced AA2017 composite powder produced by high-energy ball milling (HEBM) was investigated. AA2017 + SiC_p_ composite powder with different amounts of SiC particles (5, 10, and 15 vol%) was successfully prepared from gas-atomized AA2017 aluminum alloy powder with a particle size of <100 μm and silicon carbide (SiC) powder particles with an average particle size of <1 μm. An optical microscope (OM), X-ray diffraction (XRD), and scanning electron microscope (SEM) were utilized to characterize the microstructure of the milled composite powder at different milling periods. The results indicated that the SiC particles were homogeneously distributed in the AA2017 matrix after 5 h of HEBM time. The morphology of the particles transformed from a laminar to a nearly spherical shape, and the size of the milled powder particles reduced with increasing the content of SiC particles. The XRD analysis was carried out to characterize the phase constituents, crystallite size, and lattice strain of the composite powders at different milling periods. It was found that with increasing milling time and SiC volume fraction, the crystallite size of the aluminum alloy matrix decreased while the lattice strain increased. The average crystallite sizes were reduced from >300 nm to 68 nm, 64 nm, and 64 nm after 5 h of milling, corresponding to SiC contents of 5, 10, and 15 vol%, respectively. As a result, the lattice strain increased from 0.15% to 0.5%, which is due to significant plastic deformation during the ball milling process. XRD results showed a rapid decrease in crystallite size during the early milling phase, and the minimum grain size was achieved at a higher volume fraction of SiC particles. Microhardness tests revealed that the milling time has a greater influence on the hardness than the amount of SiC reinforcements. Therefore, the composite powder milled for 5 h showed an average microhardness three times higher than that of the unmilled powder particles.

## 1. Introduction

Currently, aluminum matrix composites (AMCs) have emerged as essential engineering materials for achieving high performance, characterized by their lightweight design, high strength, wear resistance, creep response, and favorable temperature resistance [1,2,3,4,5]. Different types of reinforcement materials, such as SiC, Al_2_O_3_, B_4_C, TiC, Si_3_N_4,_ Ti_2_B, ZrO_2_, and graphite, are commonly used in the production of aluminum metal matrix composites for various applications [6,7,8]. Ceramic particles are important to improve the properties of AMCs and are more favorable than fiber reinforcements because varying the volume fractions easily controls the microstructure and mechanical properties [9]. Among the various ceramic reinforcements, silicon carbide (SiC) and alumina (Al_2_O_3_) ceramic particles are the most commonly used reinforcements added to improve the strength of the aluminum matrix materials [10,11]. Due to its good thermodynamic and chemical stability as well as its good wettability, SiC is a widely used reinforcement particle in AMC [12]. Appropriate homogeneous distribution and interfacial bonding between aluminum and SiC reinforcement during the synthesis process lead to an increase in the overall strength of the composites [13,14].

Mechanical alloying (MA) is a powder metallurgy technique in which powder particles experience successive cycles of cold welding, fracturing, and rewelding during a high-energy ball milling process [15]. Mechanical alloying overcomes the agglomeration of reinforcement particles and enables uniform dispersion of the reinforcement particles in aluminum or other metal matrix materials [16,17]. High-energy ball milling (HEBM), commonly referred to as mechanical alloying (MA) [18], is an effective method for synthesizing composite powders with nanoscale grains and reinforcement particles with uniform distribution in the matrix [19,20,21]. In this technique, the ductile metal matrix and ceramic reinforcement particle mixture are gradually exposed to a high-energy impact in a milling container containing a high amount of milling balls. The rotation of the HEBM rotor causes collisions between the balls and the powder particles, resulting in repeated plastic deformation, cold welding, and fragmentation of the starting powders. Consequently, morphological changes occurred in the severely deformed composite powders due to the cold welding and fracturing mechanisms. Cold welding is more dominant in the early phase of the milling process and leads to an increase in the average particle size, while the fracturing process subsequently reduces the size of the composite particles. Then the welding and fragmentation events reach a steady-state condition after a sufficient milling time [22,23].

According to the literature, the mechanical alloying method has been successfully employed for the synthesis of aluminum matrix composites reinforced with ceramic particles. Nestler et al. [24] produced composites from AA2017 aluminum alloy reinforced with 5, 10, and 15 vol% SiC and Al_2_O_3_ through high-energy ball milling. The milling process involved adjusting the rotational speed in the range of 400 to 700 rpm for up to 4 h, with the controlled addition of stearic acid during the entire milling process. The study focused on the investigation of the distribution and embedding of the reinforcement particles in the Al alloy matrix. The results showed that the formation of the composite powders was influenced by the process parameters and the amount of stearic acid added during the milling process. In addition, the milled composite powders were sintered at 450 °C and then extruded into rods at 350 °C. It was found that the AA2017 matrix with homogeneously distributed SiC or Al_2_O_3_ particles exhibited better microstructure and mechanical properties than the unreinforced AA2017 alloy. Wang et al. [25] studied the influence of SiC contents and T6 treatment on the mechanical properties of SiC_p_/Al6061 composites, which were produced through ball milling and hot pressing sintering. The results revealed that the SiC particles achieved a uniform dispersion in the 6061Al matrix after 10 h of milling. The study reported an increase in the tensile strength of the composites and a reduction in elongation with higher SiC particle content. Salem et al. [26] examined the influence of the Al matrix, SiC reinforcement sizes, and SiC content on the microstructural, physical, and mechanical properties of Al-SiC composites. The study revealed that the size of SiC particles has a greater effect on the electrical resistivity, thermal conductivity, and microhardness of the composites compared to the amount of SiC reinforcement. Salur et al. [27] examined the mechanical properties of AA7075 composites reinforced with titanium carbide nanoparticles (5 wt% TiC_NP_) prepared by mechanical alloying. The results revealed that the hardness value was three times higher than the initial AA7075 alloy after 10 h of milling due to the homogeneous distribution of TiC nanoparticles in the matrix. Prashanth et al. [28] investigated the influence of milling time and volume percentage of Al_2_O_3_ on the morphology, grain refinement, and mechanical properties of AA7017-Al_2_O_3_ nanocomposites. It was found that the variation of the morphology of the particles and the uniform dispersion of the Al_2_O_3_ reinforcement led to an improvement in the hardness and compressive strength of the composite. Most of the previous studies focused on the characterization of the morphology and particle size distribution of Al + SiC_p_ composite powders, with limited discussion of XRD phase analysis [29,30,31]. However, the microstructure, structural evolution, and particularly the relationship between the crystallite size (grain size) and micro-strain in AA2017 + SiC_p_ composite powders have not been fully studied.

In this study, the AA2017 + SiC_p_ composite powder with different amounts of SiC particles (5, 10, and 15 vol%) were prepared by high-energy ball milling. Therefore, the main objective of the study was to investigate the effect of milling time and volume fraction of SiC reinforcement on the morphology, microstructure, variation of crystallite size, and lattice strain, as well as the mechanical behavior of the composite powder.

## 2. Materials and Methods

### 2.1. Raw Materials

**Figure 1:**
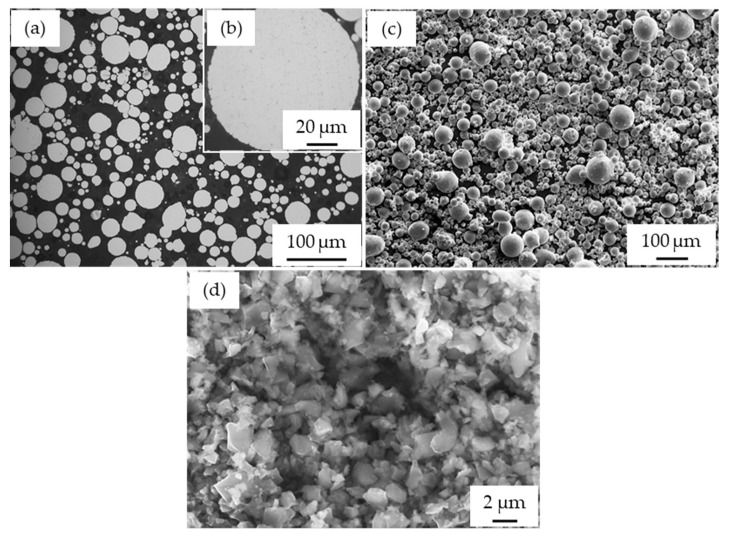
Micrographs of the as-received powders: (**a**,**b**) optical microscope images of the cross-section of AA2017 aluminum alloy; (**c**) SEM morphology of AA2017; and (**d**) SEM image of SiC.

Gas-atomized AA2017 aluminum alloy powders, with a particle size of <100 μm supplied from TLS Technik GmbH (Bitterfeld-Wolfen, Germany), were used as the matrix material. The chemical composition (wt%) of the gas-atomized AA2017 alloy is 4.1% Cu, 0.7% Mg, 0.8% Mn, 0.2% Fe, 0.1% Si, and balanced Al [32]. Silicon carbide (SiC) powder particles with an average particle size of <1 μm supplied by ESK-SiC GmbH (Frechen, Germany) were used as reinforcement material. The exact chemical composition according to the manufacturer’s data sheet is presented in Table 1. The microscopic images of the AA2017 powder (Figure 1a–c) show a spherical morphology, while the reinforcing SiC material has a polygonal shape (Figure 1d).

### 2.2. Composites Powder Preparation

The milling process was carried out in a high-energy ball mill Simoloyer CM08 (Zoz GmbH, Wenden, Germany). During the ball milling process, G600 AISI 420C stainless steel balls (Ø 4.76 mm) were used with a ball-to-powder ratio (BPR) of 10:1. A precise digital weighing balance (Mettler Toledo, Kern & Sohn GmbH, Balingen, Germany) was used for the accurate weighing of the individual starting powders with the required volume fraction. Before the milling process, the powder mixtures were blended in a Turbula System Schatz (Willy A. Bachofen AG Maschinenfabrik, Basel, Switzerland) for 15 min. The Simooyer CM08 has a horizontally arranged rotor that accelerates the balls which collide or rub against each other. Ball collisions cause fragmentation and cold welding on the composite particles [15]. Therefore, 0.5 wt% of stearic acid (C_18_H_36_O_2_) was utilized as a process control agent (PCA) to manage the undesirable cold welding and agglomeration of the composite powder particles. The total weight of the powder mixture was 800 g, and the capacity of the milling tank was 5 L. The ball milling process was performed in the closed container under a residual air atmosphere. The milling speed was cyclically varied between 400 and 600 rpm (5 min at 400 min^−1^ and 5 min at 600 min^−1^) for different milling times up to 5 h. The cyclic method reduced cold welding and agglomeration between particles [33,34]. The milling process was interrupted at the time intervals of 0.5, 1, 2, 3, 4, and 5 h to take out a small amount of powder for microstructural and mechanical property analysis. The mechanical alloying was implemented to attain a good distribution of SiC reinforcement with different volume percentages (5, 10, and 15 vol%) in the AA2017 matrix. The parameters used for the high-energy milling process are summarized below in Table 2.

### 2.3. Composite Powder Characterization

The microstructures of the composite powder samples at different milling times were characterized using optical microscopy (Olympus GX51F, Tokyo, Japan) equipped with Olympus stream motion software 2.4.3. The morphological characteristics of the as-received powders and the ball-milled composite powders were further analyzed by scanning electron microscopy (LEO Electron Microscopy Ltd., Leo 1455VP, Cambridge, UK) using both backscattered electron (BSE) and secondary electron (SE) imaging modes.

The composite powder samples were cold-mounted in an epoxy resin and hardener mixture to prepare metallographic samples for microscope characterization. The resin-to-hardener weight ratio of 2:0.9 was used. Subsequently, the mounted samples were allowed to rest at room temperature for a minimum of 8 h to ensure complete hardening of the epoxy resin. Then, the mounted samples were ground step by step in increasing order of the grinding sheet grades. The grinding process was performed using SiC papers with grit numbers of 120, 240, 400, 600, 1000, and 2500, respectively, under a flow of water. After the grinding steps, the samples were polished using 6, 3, and 1 µm polishing plates, respectively, with diamond suspensions.

The microhardness of the powder particle samples was measured using a Tukon 1102 Wilson^®^ hardness tester (Illinois Tool Works Test & Measurement, Shanghai, China) equipped with Buehler OmniMet MHT software 7.4. Prior to the microhardness measurement, the composite powder samples underwent preparation involving cold mounting with epoxy resin, followed by a grinding and polishing process to achieve a flat and smooth surface. Subsequently, the measurements were conducted on the polished surface of the powder particles with an indentation load of 10 g force (gf) (HV0.01) and a holding time of 10 s. The microhardness testing device’s indenter has a square pyramid shape made of diamond with an angle of 136° between the two opposing sides. During the measurement, the force was gradually applied to the specimen’s surface, leading to the creation of a rhombus-shaped indentation region. After removing the load, the indentation region was magnified using the optical lens system integrated into the hardness tester apparatus, with a magnification of 50× to determine the diagonals. Then, with the help of the connected computer, both diagonal lengths of each indentation were measured from the magnified image, and the OmniMet MHT software immediately calculated the statistical values of the diagonal lengths. The microhardness (HV) is then calculated using the following equation [35].

(1) $$\mathrm{HV}=1.8544\frac{F}{d^{2}}$$

where F is the applied load measured in gf and d is the diagonal length of the indentation in mm. 15 measurements were taken for each sample to calculate the average value and standard deviation of the measurements.

X-ray diffraction analysis (XRD) was carried out to measure the phase composition and crystallite size of the composite powders at various milling times and reinforcement content. The samples were measured using a D8 Discover X-ray diffractometer from Bruker AXS with Co-Kα-radiation (*λ =* 0.178897 nm) at an operating voltage of 40 kV and current of 40 mA with point focus, a 1 mm collimator, and a LynxEye XE-T detector. The measurement time per sample was 8.3 h. The XRD pattern diffraction angle was recorded in the 2θ range between 20 and 130°. The quantitative phase analysis of the measured data was analyzed using TOPAS V5 software (Bruker AXS, Karlsruhe, Germany). The crystallite size and lattice strain of the composite powder particles are determined through XRD peak broadening by using the William and Hall method according to the following equation:

(2) $$\beta \cos\theta =\frac{0.9\lambda }{D}+2\varepsilon \sin\theta$$

where β is the full width at half maximum intensity (FWHM) of the diffraction peak, θ is the Bragg angle, λ is the wavelength of the X-ray, D is the crystallite size, and ε is the lattice strain of the composite powders [36].

Miller indices (hkl) are used to observe different atomic levels, and the identified diffraction peaks can be associated with the atomic levels to assist in the analysis of the microstructure of the powder milled at different milling times [37].

## 3. Results and Discussion

### 3.1. Microstructural Characterization

**Figure 2:**
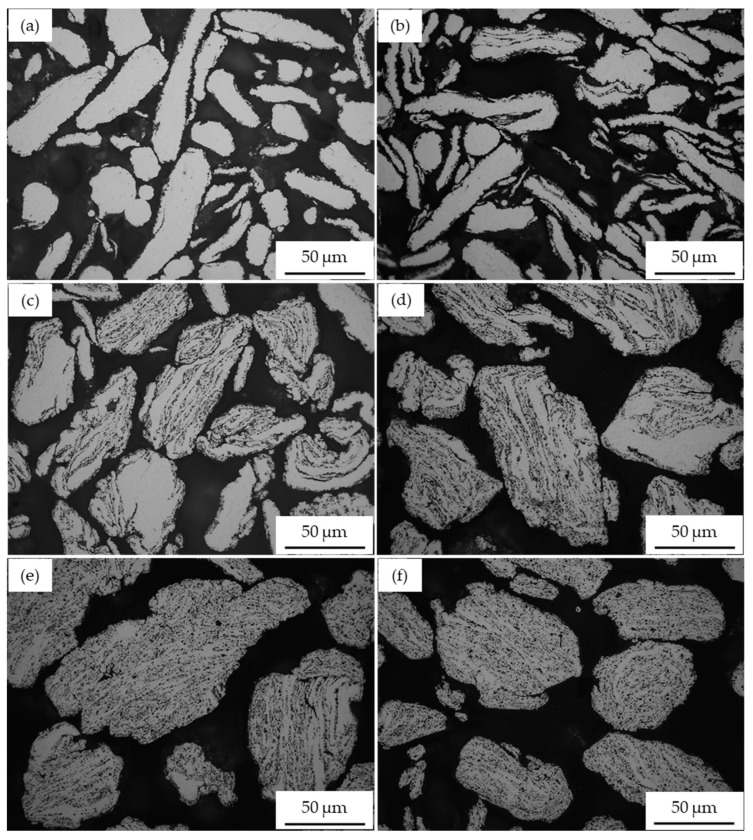
Optical micrographs of AA2017 + 5 vol% SiC_p_ powders milled for different times: (**a**) 0.5 h; (**b**) 1 h; (**c**) 2 h; (**d**) 3 h; (**e**) 4 h; and (**f**) 5 h.

Figure 2 illustrates the optical microscopy images of AA2017 + 5 vol% SiC_p_ composite powders high-energy ball milled at different times. It can be observed that various types of microstructural changes occurred in the composite powder during the milling process. In the early stage of the milling process, the initial spherical aluminum alloy powder particles were deformed into a flattened shape (Figure 2a). At this stage, the SiC particles did not start to deposit on the flattened surface of the aluminum alloy particles. Up to 1 h of the milling period, the powder exhibits minimal effects of cold welding, fracturing, and rewelding characteristics (Figure 2b). It can also be observed that the influence of the reinforcing particles is not noticeable at this stage. Increasing the milling time to 2 h initiated cold welding of the particles, and the particles became more flattened and acquired flake-like shapes due to the intense collision forces between the balls-powders-balls in the milling container (Figure 2c). It is evident that at this stage, the reinforcement particles begin to settle around the surface of the flakes. These obtained particles contained a mixture of reinforced and unreinforced areas. Thus, the areas with SiC particles appear as black spots, while the areas without reinforcement have smooth and bright surfaces. Certainly, the flake surface of the particles provides free space for the SiC dispersion and contributes to a homogeneous distribution in the AA2017 alloy matrix. Further increasing the milling time enhanced the attachment of SiC particles to the matrix. However, the cold welding effect becomes more dominant, and the flattened particles laminated together and formed larger particles (Figure 2d). During the high-energy ball milling, the powder particles were repeatedly cold welded and fractured, which can lead to strong interfacial bonding between the matrix and the reinforcing particles. This is an expected process for ductile aluminum materials and leads to improved mechanical properties of the final composite powder [38]. However, it is essential to maintain the cold welding and fracture mechanisms until the equilibrium state is reached. Stearic acid was applied every hour to control the excess amount of cold welding and adhesion of particles during the milling process.

Figure 2e shows the dispersion of SiC_p_ within the aluminum particles after 4 h of milling. It can be observed that the reinforcement particles are nearly evenly distributed within the AA2017 alloy matrix. However, even after a long milling time, there are still a few areas free of SiC particles. At this milling duration, the welding of flattened particles leads to an increase in the particle size of the composite powder, resulting in the formation of larger and coarser particles. Similar observations were reported in the literature for the milling of particle-reinforced Al composites after 4 h of milling [24,34]. The dispersion of SiC particles in the AA2017 matrix progressively improved with increasing milling time until a uniform distribution was achieved. As illustrated in Figure 2f, homogeneous dispersion of SiC particles in the Al matrix was accomplished after 5 h of HEBM time.

**Figure 3:**
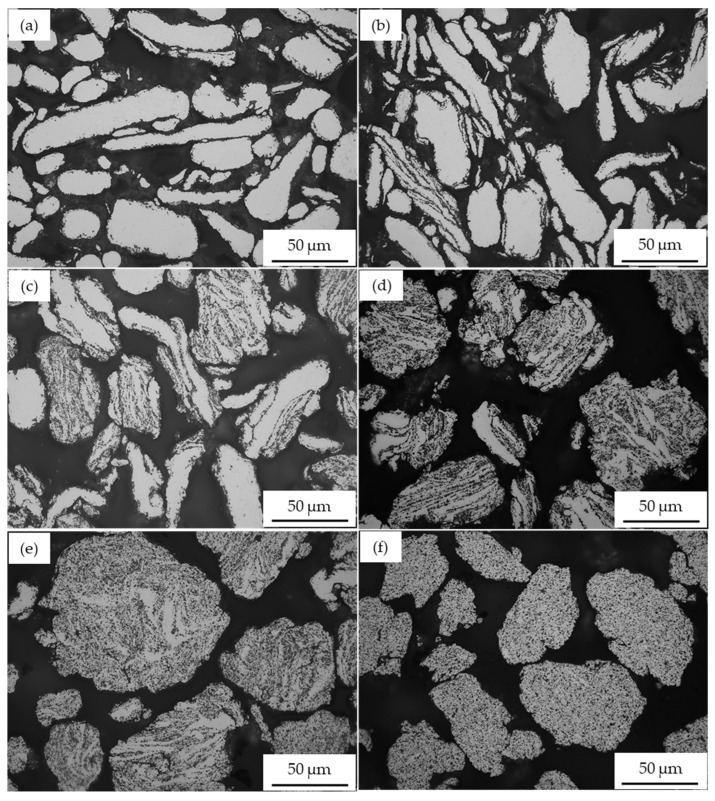
Optical micrographs of AA2017 + 10 vol% SiC_p_ powders milled for different times: (**a**) 0.5 h; (**b**) 1 h; (**c**) 2 h; (**d**) 3 h; (**e**) 4 h; and (**f**) 5 h.

Figure 3 shows the morphology of a high-energy ball-milled A2017 + 10 vol% SiC_p_ composite powder. In the initial milling phase, the Al alloy particles were transformed into a flake-like shape without the deposition of SiC particles, as shown in Figure 3a,b. This is consistent with the behavior observed in the AA2017 + 5 vol% SiC_p_ composite powder. Extension of the milling time up to 3 h resulted in a change in the morphology of the particles from a flattened to a laminar shape (Figure 3c,d). Simultaneously, the SiC particles were deposited on the surface of the matrix. After 4 h of milling, it can be observed that the laminar tendency of the particles decreased, and spherical particles were obtained. However, these particles are larger as the cold welding mechanisms were predominant during milling (Figure 3e). At this stage, the SiC reinforcement particles are nearly evenly distributed within the AA2017 alloy matrix. As the milling time extended up to 5 h, a reduction in particle size and a uniform distribution of SiC particles were achieved (Figure 3f). Consequently, a significant reduction in particle size was achieved due to the dominance of the fracture mechanism during the milling process. 

**Figure 4:**
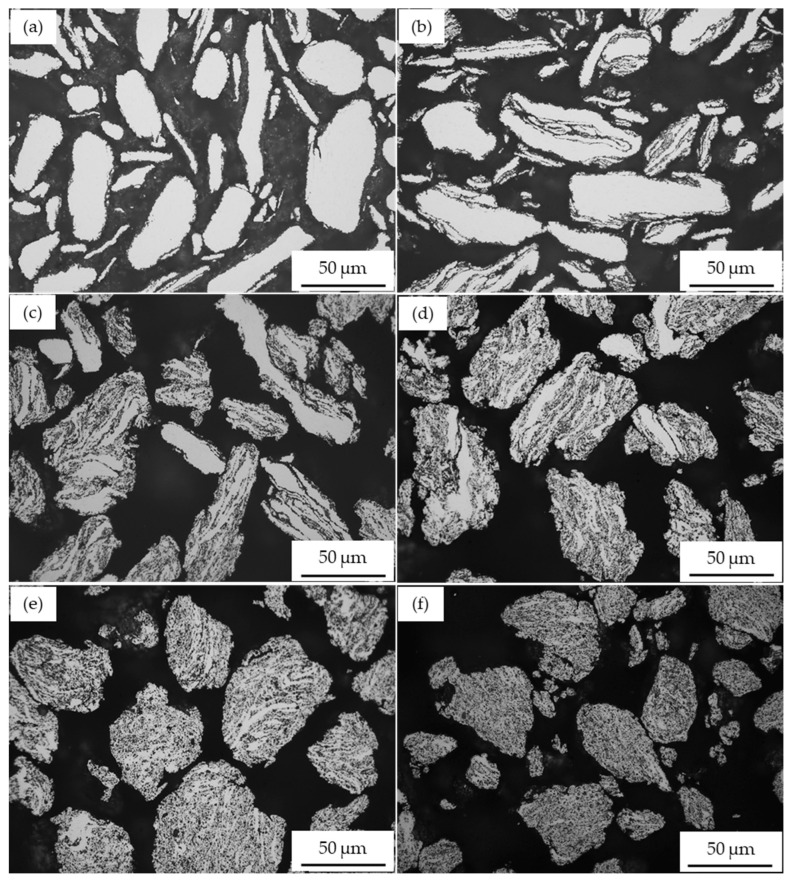
Optical micrographs of AA2017 + 15 vol% SiC_p_ powders milled for different times: (**a**) 0.5 h; (**b**) 1 h; (**c**) 2 h; (**d**) 3 h; (**e**) 4 h; and (**f**) 5 h.

The effect of milling time on the morphology and microstructure of A2017 + 15 vol% SiC_p_ composite powder is presented in Figure 4. The milling phases follow a similar sequence as observed in A2017 + 5 vol% SiC_p_ and A2017 + 10 vol% SiC_p_ composite powders. The results show that the influence of the SiC particles was not noticeable after a short milling time of up to 1 h (Figure 4a,b). With an increase in milling time to 2 h, the reinforcing particles begin to deposit noticeably on the flattened surface of the matrix, as shown in Figure 4c. The dispersion of SiC particles in the AA2017 alloy matrix increases with increasing milling time (Figure 4d,e). Moreover, the higher volume fraction of SiC particles enhances the fracture mechanism within the Al alloy matrix, which is favored by the presence of reinforcing particles during the ball milling process. As shown in Figure 4f, nearly equiaxed particles with a homogeneous distribution of SiC particles are achieved after 5 h of HEBM.

Achieving a uniform distribution is essential after milling to enhance the mechanical properties of the aluminum alloy matrix with SiC reinforcement. The dispersion of SiC particles within the AA2017 matrix intensifies as the milling time increases, eventually reaching a state of homogeneous distribution. Homogeneous distribution of SiC particles in the Al matrix was achieved after 5 h of HEBM for different amounts of reinforcement (5, 10, and 15 vol% SiC). Therefore, a high-energy ball milling method can achieve homogeneous dispersion of SiC particles in the AA2017 alloy matrix.

**Figure 5:**
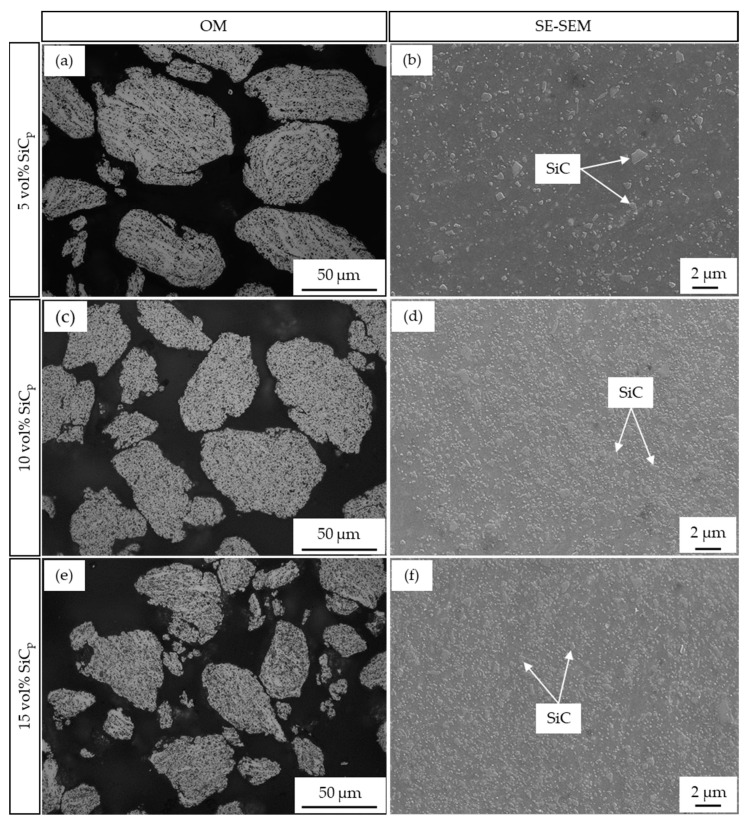
AA2017 + SiC_p_ composite powders containing 5, 10, and 15 vol% SiC particles after 5 h of HEBM: (**a**,**c**,**e**) optical microscope; (**b**,**d**,**f**) SE-SEM micrographs.

Figure 5 shows the OM and SEM micrographs of the AA2017 + SiC_p_ composite powders containing 5, 10, and 15 vol% SiC_P_ after 5 h of milling. The composite powders prepared with different amounts of reinforcement showed similar microstructural changes in the initial and progressive stages of milling time. However, the disposition of a higher number of particles on the matrix materials was observed for a higher content of SiC reinforcement (Figure 5d,f). When the volume fraction of SiC_p_ increases, the distribution of the SiC particles in the matrix becomes denser, as can be clearly shown in Figure 5. Hence, these reinforcing particles might accelerate the premature fracture of the matrix particles and serve as a milling medium during the milling process.

**Figure 6:**
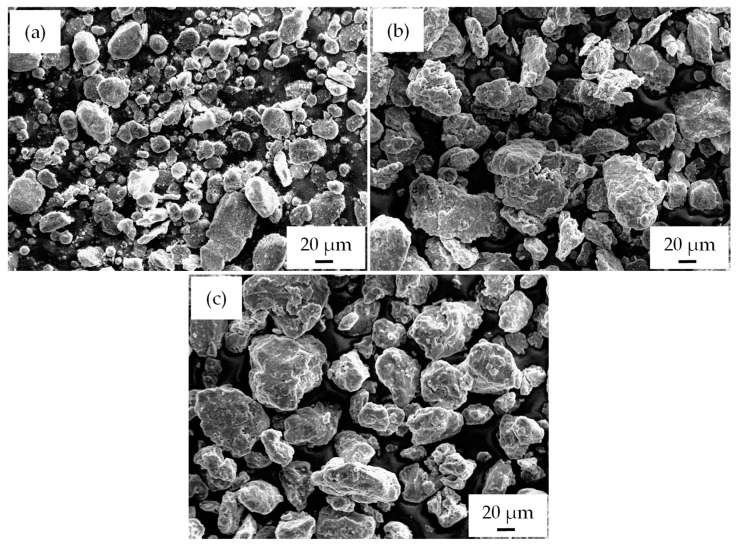
SEM morphologies of AA2017 + 10 vol% SiC_p_ after (**a**) 0.5 h; (**b**) 2 h; and (**c**) 5 h of HEBM time.

Figure 6 shows the SEM morphology of AA2017 + 10 vol% SiC_p_ composite powders, illustrating the changes in morphology corresponding to the different durations of ball milling. As the milling time increases, the shape of the particles changes due to the cold welding and fracturing of the powder particles. Cold welding leads to an increase in particle size, while the fracture mechanism reduces the size. Up to a milling time of 2 h, the powders remain in a soft state, with cold welding dominating the process. As presented in Figure 6a,b, the particle size increased and flake-like particles were formed due to the effects of cold welding and particle deformation. The shape of the particles changed from a laminar type of morphology to a nearly spherical shape after 5 h of milling, as shown in Figure 6c. In contrast to the initial stages of milling, the particle size is slightly reduced due to the work-hardening of the particles, and the fracture rate dominates as the milling time increases. Nevertheless, a noticeable reduction in particle size was not achieved after a longer milling time. This indicates that the fracture and cold welding processes have not yet reached a steady state after 5 h of milling time. The milling processes were interrupted after 5 h of milling to protect the powders from excessive cold welding and the build-up of particles in the milling media.

### 3.2. Phase Analysis of the AA2017 + SiC_p_ Composite Powders

**Figure 7:**
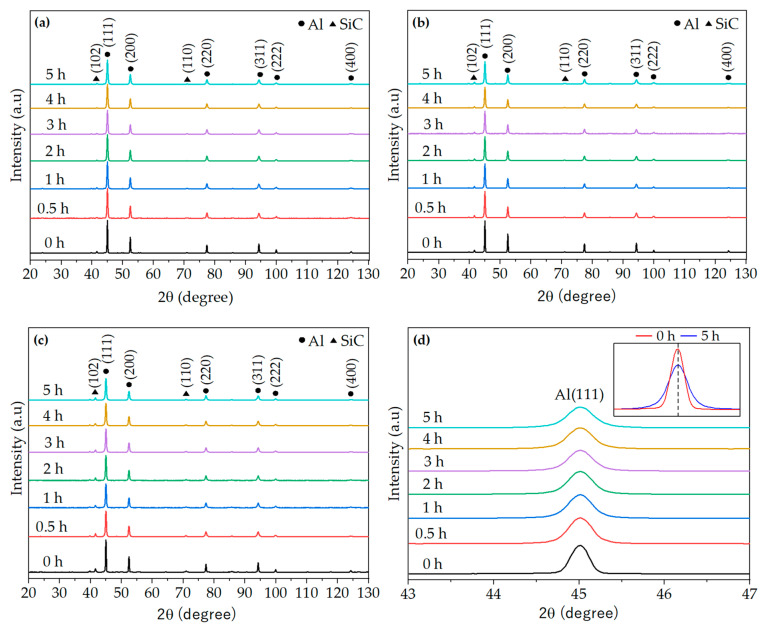
XRD patterns of AA2017 with (**a**) 5, (**b**) 10, and (**c**) 15 vol% SiC_p_ composite powders after different milling times; (**d**) Magnified view of the main peaks of the AA2017 + 5 vol% SiC_p_ composite powders.

Figure 7 shows the comparison of XRD patterns of AA2017 + SiC_p_ composite powders with different SiC contents recorded after various milling times. Figure 7a,b,c illustrate the XRD patterns of milled powders corresponding to 5, 10, and 15 vol% SiC_p_, respectively. Figure 7d illustrates the enlarged view of the main peaks Al (111) of the AA2017 + 5 vol% SiC_p_ composite powders. In all XRD diffraction patterns of the milled composite powders, six distinct diffraction peaks corresponding to the FCC structure of aluminum, along with minor SiC peaks, were identified. Hence, both cubic Al and hexagonal 6H-SiC phases were detected in all XRD patterns of the milled composite powders. As can be seen in Figure 7a–c, only a few diffraction peaks with a lower intensity of SiC phases are visible in the XRD patterns. This occurrence could be attributed to the low volume fraction of SiC phases and/or the fine particle size of the reinforcement in the AA2017 + SiC_p_ powder mixture [39]. In addition, the XRD pattern of the milled powders confirmed that no new peak existed in all XRD patterns, and there was no reaction between Al alloy and SiC particles for various milling times as Al and SiC phase peaks were still present in the composite powders. This indicates that no new phases were formed throughout the milling process. Similar findings were reported in the literature [13,31,40].

The magnified view of Figure 7d shows the position (i.e., 2 theta angles) of the intense peak Al (111) for all milling periods. All peaks are not noticeably shifted to the higher and/or lower values of the diffraction angles with increasing milling time. This suggests that the alloying elements and impurities were not dissolved into the lattice of the aluminum matrix during the HEBM process. Many authors previously reported that the shift in the XRD peak positions is associated with the dissolving of smaller matrix alloying elements, particularly impurities such as iron, and reinforcing particles within the lattice of the aluminum alloy matrix phase during the milling process [34,41,42]. As shown in the right corner of Figure 7d, the first peak of the Al diffraction plane (111) in AA2017 + 5 vol% SiC_p_ is reduced, and its width is significantly broadened after 5 h of milling. The intensity of the Al peaks gradually reduced, and the full width at half maximum (FWHM) broadened as the milling time increased up to 5 h (Figure 7d). This reduction in peak height and broadening confirms the refinement of grain size and the accumulation of lattice strain in the particles of the composite powder during the mechanical alloying process. However, the peak intensity and FWHM of SiC_p_ peaks have not changed significantly.

**Figure 8:**
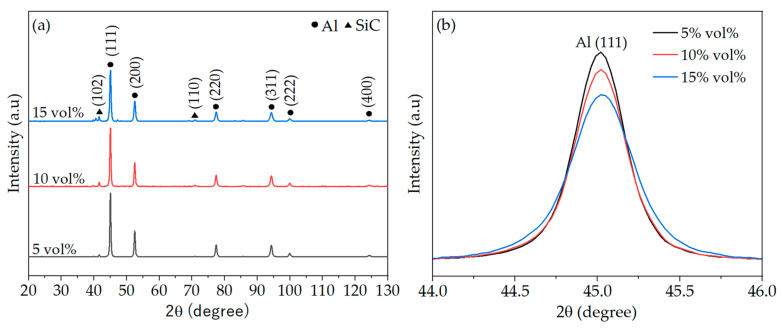
XRD patterns of AA2017 with (**a**) 5, 10, 15 vol% SiC_p_ composite powder after 5 h of HEBM; (**b**) Magnified view of the main peaks Al (111) of the 5, 10, 15 vol% SiC_p_ composite powder after 5 h of HEBM.

Figure 8 shows the comparison of the XRD patterns of AA2017 + SiC_p_ composite powder with different SiC contents after 5 h of milling. Figure 8a illustrates the XRD patterns of AA2017 with 5, 10, and 15 vol% SiC_p_ composite powder, respectively, and Figure 8b shows the magnified view of the main peaks of Al (111) after 5 h of milling. It is worth mentioning that no additional peaks with other phases were observed at different SiC contents after 5 h of milling, which indicates that there was no reaction between the matrix and the SiC particles during the ball milling, as shown in Figure 8a. As shown in Figure 8b, the intensity of the Al peaks decreases and the peak width broadens with increasing the volume fraction of SiC reinforcement. The intensity of the main Al peak (111) decreased and the peak width broadened with increasing the volume fraction of SiC reinforcement, as illustrated in Figure 8b. This broadening of the XRD peaks indicates grain refinement and an accumulation of lattice strain in the composite powder particles as the volume fraction of the reinforcement content increases. According to the literature, the smaller grain size of the powder particles results in lower peak heights and broadened peak intensities [1,39,43].

**Figure 9:**
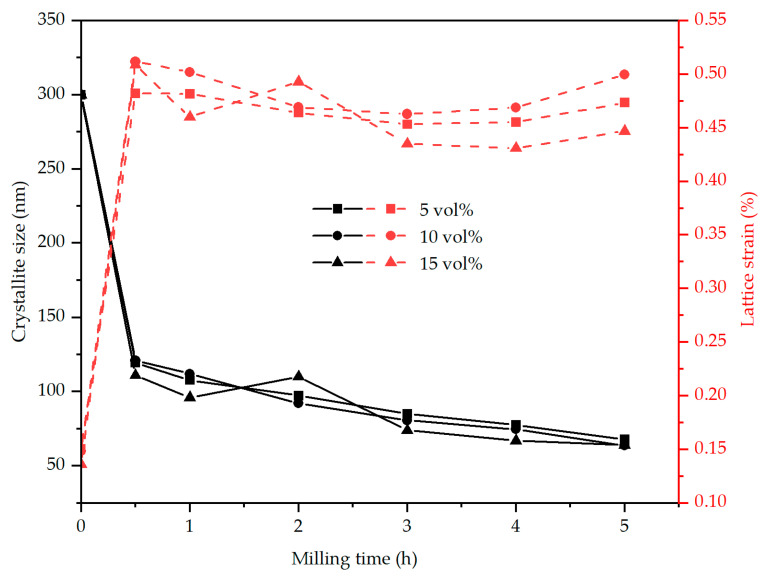
Crystallite size and lattice strain of AA2017 + 5 vol% SiC_p_, AA2017 + 10 vol% SiC_p_, and AA2017 + 15 vol% SiC_p_ composite powders at different ball milling times.

The influence of milling time on the crystallite size and lattice strain of AA2017 + SiC_p_ composite powder with various amounts of SiC reinforcement is presented in Figure 9. With the extension of milling time and an increase in SiC volume fraction, a clear reciprocal relationship between the crystallite size and lattice strain becomes noticeable. It can be seen from Figure 9 that the crystallite size of the particles reduces with increasing the ball milling time and SiC content. As a result, the average crystallite sizes of the aluminum alloy particles decrease from >300 nm to 68 nm, 64 nm, and 64 nm, respectively, corresponding to SiC contents of 5, 10, and 15 vol%, respectively, after 5 h of milling time. It is also worth mentioning that the crystallite size decreases rapidly up to 0.5 h to about 119, 121, and 111 nm for 5, 10, and 15 vol% SiC_p_ composite powder, respectively, and that a slow crystallite (grain) size change occurs between 0.5 and 5 h. It is clear that the crystallite sizes were reduced rapidly in the initial phase of the milling period. In addition, the lattice strain increased from <0.15% to about 0.47%, 0.50%, and 0.45% for 5, 10, and 15 vol% SiC_p_ composite powders, respectively, after 5 h of milling (Figure 9). Lattice strain increased rapidly in the early stage of milling up to 0.5 h, and further milling up to 5 h had no significant effect on the micro-strain level. It was also worth noting that the lattice strain increased slightly with increasing volume fractions of reinforcements but decreased at a higher volume fraction (15 vol% SiC_p_) after 2 h of milling. However, the sample AA2017 + 15 vol% SiC_p_ provided a better result, as a smaller crystallite size was achieved even with a low lattice strain. In general, the amount of SiC reinforcement added to the AA2017 alloy appears to be ineffective in inducing significant changes in crystallite size and lattice strain. However, HEBM was more effective in reducing crystallite size than increasing SiC content. The results are consistent with previous findings by [40,44].

The reduction of the grain size and the accumulation of lattice stresses are caused by severe plastic deformation, cold welding, and fracture of the powder particles during the mechanical alloying process [15,45]. Crystal imperfections, including dislocations and point defects, increase due to the strong deformation of composite powder particles during the milling process [33,41,44]. The defects increase the internal energy of the lattice strain, and the crystal structure becomes unstable. In the early stage of the milling period, the low-angle sub-boundaries are formed due to the relocation of the dislocations to a lower energy state. Then, longer milling times lead to more dislocations and high plastic deformations, which enhance the misorientations between sub-grains at their boundaries. Eventually, the lower angle sub-boundaries progressively transform into the higher angle, and ultrafine grains with sizes in the nano-range are formed [41,43]. In addition, the presence of non-deformable and hard SiC particles and the interaction with dislocations accelerate the grain refinement process. Hard-reinforcing particles hinder the motion of dislocations, leading to a rise in dislocation density. On the other side, the addition of small size (<1 µm) SiC particles leads to dislocation multiplication due to the Orowan bowing mechanism [34,39,42]. This means that a stronger interaction between SiC particles and dislocations causes a higher degree of dislocation and an accumulation of micro-strains, which consequently contributes to a decrease in crystallite size (grain refinement) in the composite powder particles.

**Figure 10:**
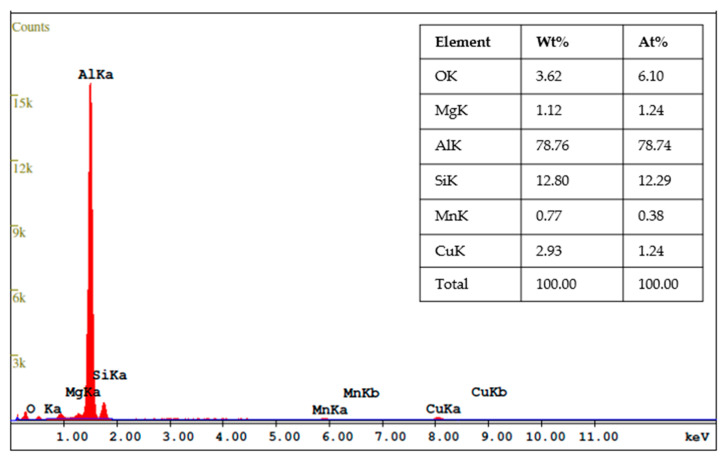
EDX spectrum of AA2017 + 10 vol% SiC_p_ composite powder after 5 h milling time.

The energy dispersive x-ray analysis (EDX) analysis of AA2017 + 10 vol% SiC_p_ composite powder after 5 h of ball milling time is presented in Figure 10. The spectrum shows noticeable peaks corresponding to elements such as aluminum (Al), copper (Cu), magnesium (Mg), manganese (Mn), silicon (Si), and oxygen (O_2_), which confirmed the presence of these elements in the composite powder. It is noteworthy that the EDX analysis did not reveal the formation of new phases in the composite, except for the 3.62 wt% of oxygen. It is considered that the detected oxygen peak comes from the residual air from the closed chamber during the milling process.

### 3.3. Microhardness Measurement

**Figure 11:**
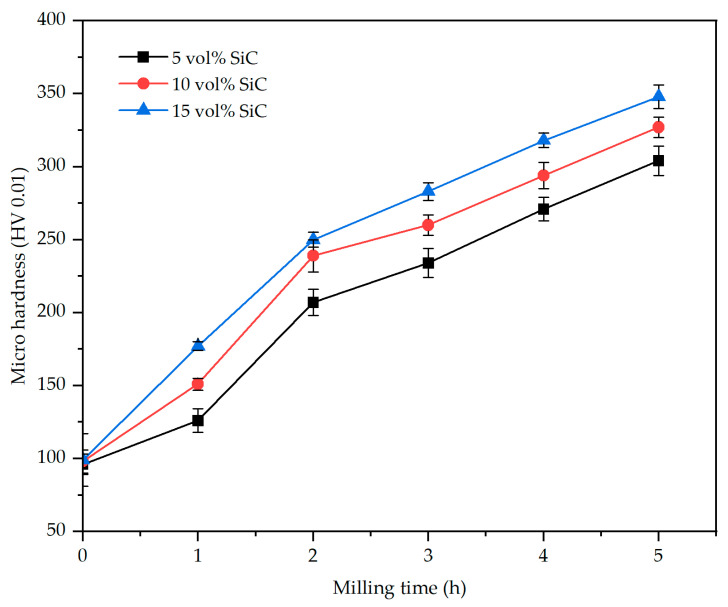
Variation of microhardness with different milling times and reinforcement volume fractions of AA2017 + SiC_p_ composite powder.

Figure 11 illustrates the variations in microhardness of the AA2017 + SiC_p_ composite powder in relation to milling time and SiC reinforcement amount. It is noticeable that the microhardness of composite powder samples increased continuously as the milling time and reinforcement content increased. This is due to the fact that increasing the milling time enhances and accelerates the work-hardening and fracture of the powder particles, which leads to an increase in microhardness values [46,47]. The existence of SiC particles in the AA2017 alloy matrix can potentially increase the deformation and the work-hardening of the powder particles. Moreover, the hard reinforcing particles facilitate deformation in the matrix and improve the cold welding and fracture mechanisms because of their hard and non-deformable properties. Therefore, it helps in enhancing dislocations and grain refinement, subsequently leading to an increase in microhardness values. Increasing the reinforcement content accelerates the work-hardening and fracture mechanisms of the matrix. This is attributed to the SiC particles functioning as a strengthening medium, hindering dislocation movement, and improving load transfer within the Al alloy matrix. In the present study, it was found that the average microhardness of the composite powder increased from ∼96 HV before ball milling to 304 (±10), 327 (±10), and 348 (±18) HV, corresponding to SiC content of 5, 10, and 15 vol%, respectively, measured at the load of 10 gf (HV0.01) after 5 h of HEBM time (Figure 11). It is seen that the hardness of the composites increases slightly with an increasing volume fraction of SiC particles. The maximum microhardness value of 348 HV was obtained for AA2017 + 15 vol% SiC_p_ composite powder milled for 5 h. This hardness value is three times greater than the unmilled powder. However, the influence of the ball milling time on hardness is more significant than the SiC volume fraction. The results are consistent with previous studies showing that hardness increases with increasing milling time [26,29,40].

## 4. Conclusions

In this work, the effect of milling time and volume fraction of reinforcement on the morphology, microstructural, and mechanical behavior of SiC_p_-reinforced AA2017 composite powders fabricated by the HEBM process was studied. It was found that the duration of HEBM and the SiC reinforcement content exhibited remarkable effects on the morphology, grain size, and microhardness of the milled composite powder. In the early phase of milling, the originally spherical particles were transformed into flaky shapes by subsequent cold welding and fracturing mechanisms. It was observed that the SiC particles began to deposit on the deformed surface of the aluminum alloy particles after 2 h of milling. The improved microstructural and mechanical behaviors of the composite powder are attributed to the extension of the milling time, where a homogeneous distribution of SiC particles within the AA2017 alloy was obtained after 5 h of milling. With the increase in milling time, the crystallite size decreased to about 64 nm, and the lattice strain increased to 0.50% as a result of the severe plastic deformation and dislocations in the powder particles. The microhardness of the composite powders was also found to increase with the increase in milling time and showed a maximum value three times higher than that of the unmilled powder. The microhardness of the AA2017 + 15 vol% SiC_p_ composite powder, measured at a load of 10 gf (HV0.01), reached 348 HV after 5 h of milling. Compared to the microhardness value of the unmilled AA2017 powder of 96 HV, the addition of 15 vol% SiC_p_ to AA2017 showed a higher improvement in microhardness value. Therefore, the microhardness of the milled AA2017 + SiC_p_ composite powder increased due to the dispersion of the SiC particles, the work-hardening, and grain refinement during the ball milling process.

## Figures and Tables

**Table 1:** The chemical composition of the AA2017 powder used according to the manufacturer’s specifications (wt%).

**Chem. Element**	Cu	Mn	Mg	Fe	Si	Al
**Content (wt%)**	3.94	0.59	0.77	0.2	0.1	Balanced

**Table 2:** High-energy ball milling process parameters.

Milling Parameters	Value
Milling time	5 h
Milling Rotor speed	400 and 600 rpm (cyclic)
Weight of powder mixture	800 g
Weight of steel balls	8 kg
Milling ball diameter	Ø 4.76 mm
Stearic acid (PCA) amount	0.5 wt%

## Data Availability

The data that support the findings of this study are available from the corresponding author upon reasonable request.
